# T‐Cup: A Cheap, Rapid, and Simple Home Device for Isothermal Nucleic Acid Amplification

**DOI:** 10.1002/gch2.202100078

**Published:** 2021-12-26

**Authors:** Aldrik H. Velders, Michel Ossendrijver, Bart J. F. Keijser, Vittorio Saggiomo

**Affiliations:** ^1^ Laboratory of BioNanoTechnology Wageningen University P.O. Box 8038 Wageningen 6700EK The Netherlands; ^2^ TNO Microbiology and Systems Biology P.O. Box 360 Zeist 3700 AJ The Netherlands

**Keywords:** isothermal amplification, LAMP, NINA devices, phase change materials, SARS‐CoV2

## Abstract

During the SARS‐CoV2 pandemic, it has become clear that centralized testing suffers from multiple bottlenecks. Logistics, number of machines, and people available to run the diagnostic tests are limited. A solution to those bottlenecks would be a fully decentralized system, where people can test themselves at home and only report back the outcome of the test in a centralized database. Here a noninstrumental device capable of achieving isothermal conditions useful for detecting the SARS‐CoV2 RNA using loop mediated amplification (LAMP) tests is presented. This device, compared to others reported in literature or present on the market, is cheap, easy to produce and use, and has little impact on the environment. Using a simple aluminum coffee capsule, a phase change material, and a 3D printed holder, this device, when placed in boiling water, is able to maintain a temperature of 65 °C for 25 min, required for running the LAMP reaction. In principle, this device can be applied to any LAMP reaction, and hence employed for many different applications, and can be deployed in large quantities in short amount of time.

## Graphical abstract

A noninstrumental device capable of achieving isothermal conditions useful for detecting nucleic acids using loop mediated amplification is here presented. This device is cheap, easy to produce and use, and has little waste impact on the environment. The device is composed of a simple aluminum coffee capsule, a phase change material, and a 3D printed holder.

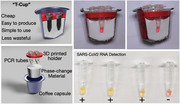

## 1 Introduction

The COVID‐19 pandemic^[^ ^1^ ^]^ has dramatically impacted our daily lives and revealed the importance of rapid, repetitive and thorough testing of large numbers of people. Effective testing, tracing, and isolation allows control over virus spread in population. Screening of about 10% of the population on regular basis has been suggested to allow control over the spread of the virus in society,^[^ ^2^ ^]^ although this percentage and the results of the simulation are still under debate.^[^ ^3^ ^]^

Whereas centralized testing facilities have been set up in relatively short amounts of time, focused on testing individuals with symptoms, significant shortcomings and logistic bottlenecks have become apparent, from sample collection, storage and transport, to lack of personnel, materials, and instruments. Taking as an example the Netherlands, a relatively small country, testing 10% of the population would require running 1.7 million tests as fast as possible. Performing more than a million tests is simply not feasible with centralized testing alone, not even for a relatively small country with efficient infrastructure such as The Netherlands. Achieving millions of tests per day is only practically realizable in decentralized testing, using self‐performed tests, directly at the population level. Self‐performed tests are also an improvement over centralization for detecting asymptomatic infected individuals who would typically not go to testing sites.

To date, the standard test for SARS‐CoV2 has relied either on detection of specific nucleic acid using polymerase chain reaction (PCR) or antigen rapid tests.^[^ ^4^ ^]^ PCR is too complex, in particular regarding the required instrumentation and sample handling, and not robust enough to be performed by untrained personnel in a home environment, and requires specific infrastructures and devices for controlling the different temperatures necessary for the amplification. Relatively simple antigen (rapid) tests have also been exploited. These can be run in classical lateral flow assays such as the archetypical pregnancy test. However, the antigen tests, although faster than nucleic acid amplification, have a lower sensitivity as, in contrast to nucleic acid amplification, they do not perform any amplification step, and are not suitable for early phases testing. Moreover, in case of a new disease, it is easier and faster to develop new primers for nucleic acid amplification rather than develop a lateral flow assay from scratch. Another emerging detection method is the loop‐mediated amplification (LAMP),^[^ ^5^ ^]^ which allows detection of small amounts of viral RNA from oral/nasal swabs even in an early stage of the infection. PCR and LAMP are both based on selective amplification and detection of part of the viral genome using specific primers. Commercially available LAMP or PCR instruments are expensive and not easy to use by untrained personnel. While PCR is anyways too complex to be performed by untrained personnel in a home environment, LAMP has the potential to provide a robust and easy to use test set‐up required for a point of care and in‐field testing thanks to its isothermal amplification as well as the possibility of working with relatively dirty samples without the need of purify the nucleic acid before the amplification step.^[^ ^6^ ^]^ Different from PCR, LAMP amplification occurs at a single temperature, is capable to work in conjunction with simplified methods for sample pre‐processing and allows visual readout of test results. Many groups around the world, including ours, are working on making LAMP simple and robust enough to be used at home by untrained personnel for favoring a true decentralized testing.^[^ ^7^, ^8^, ^9^ ^]^

The device or instrument required to run the decentralized SARS‐CoV2 LAMP reaction requires slightly more attention for home testing than testing in a laboratory. Such LAMP device should be cheap, reusable, easy to produce in quantity of millions in a short amount of time and, preferably, resulting in minimum amounts of waste. In the end, the crucial part for a LAMP device concerns incubation of the sample at a specific temperature for a certain amount of time.

Depending on the enzymes used, LAMP typically requires temperature in the range of 60–70 °C for a fixed amount of time, typically less than 30 min, for amplifying the nucleic acids in the sample. Water temperature‐controlling instruments can be as cheap as, e.g., a sous vide, and can be used to maintain the water at constant temperature,^[^ ^10^ ^]^ however, for a robust test one should not be dependent on specific hardware nor on user skills or accuracy. A cheaper solution could be a home‐made LAMP device made by simple and open‐source electronics, for example using Arduino.^[^ ^11^ ^]^ Such devices however, although being relatively cheap and easy to use, cannot be produced in numbers of millions in a short amount of time, and they will produce an unsustainable amount of electronic waste (e‐waste).

For in‐field LAMP, and especially for remote locations where electricity may be a limiting factor, noninstrumental nucleic acid amplification (NINA) devices were developed.^[^ ^12^, ^13^ ^]^ These devices use phase change materials (PCM), substances that absorb or release energy at the phase transition temperature and so provide, for a certain amount of time, a fixed temperature when in a relatively hot, respectively cool environment. For the in‐field LAMP the heating is, e.g., driven by a chemical exothermic reaction as source of heat. These devices are cheap, easy to use, and do not require electricity. The drawbacks of these instruments are a) the vessels need to be produced in large quantities, which may be problematic, and b) more importantly, the heat source is an exothermic reaction, which makes the device not safe for being used by untrained personnel, and also adds a problem in logistics, as shipping hazardous chemicals is highly regulated. In addition to this, the chemicals for producing the exothermic reaction are disposable and need to be commercially acquired for each single reaction which increments the costs, the safety, and the waste produced by these devices. A summary of the discussed LAMP devices is given in **Table** **1**.

**Table 1:** Comparison between LAMP devices

	Cost approx. (in €)	Energy source	Scalability	e‐Waste produced
Genie II (Optigene)	>10000	Electricity/battery	No	Yes
Sous vide^[^ ^10^ ^]^	60	Electricity	No	Yes
Arduino based^[^ ^11^ ^]^	10	Electricity/battery	Yes	Yes
PCM NINA^[^ ^12^, ^13^ ^]^	10	Exothermic reaction/disposable	No	No
PCM T‐Cup (this research)	<1	Boiling water	Yes	No

**Figure 1:**
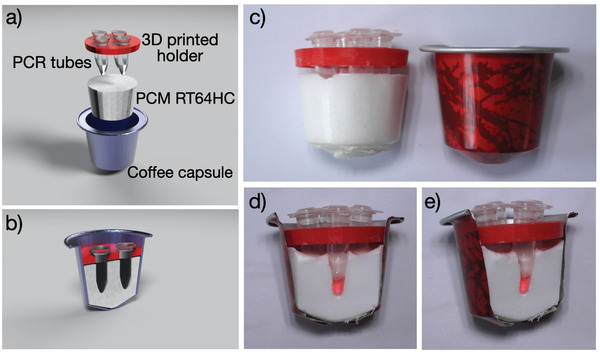
a,b) 3D render of the T‐Cup showing its components: an aluminum coffee capsule, few grams of phase change material (RT64HC), a 3D printed holder for the LAMP vials, and the PCR tubes with the reagents in them. c) Picture of the T‐Cup and d,e) picture of the cross‐section, highlighting the distance between the bottom of the vial and the aluminum capsule.

Recognizing the limitations of the different devices, we embarked on the development of a novel LAMP device which is cheap, reusable, and can be produced in large amounts in a short period of time. The device was designed such not to require chemical exothermic reactions, have limited waste produced and with a minimum cost of the device as a whole. Whereas the chemical exothermic reactions NINA designs are of relevance for in‐field measurements, for home testing one might assume most people have access to boiling water. Considering the potential large numbers needed, readily available and cheap components were chosen, such as commercially available containers (coffee capsule), PCM and 3D printable components. **Figure** **1** shows an overview of the components of the LAMP home test device which we coined temperature‐cup (T‐Cup).

## 2 Results and Discussion

In a classical NINA system, an exothermic reaction heats up a sample contained in a PCM material that will start melting at its phase changing temperature. As long as the PCM is not fully melted, the temperature remains constant. For the T‐Cup device, we decided to opt for a less invasive system by immerging the sample container with PCM into an environment with a temperature higher than the phase transition temperature, causing the PCM to melt at the constant PCM temperature.

First, we set the goal of reaching the required temperature for the corona‐LAMP (60–65 °C), then consecutively a robust method to provide a heating environment using common household equipment was searched for, then the PCM container properties and relative amounts of PCM to control temperature and time were optimized, and finally the LAMP reaction was tested, and a protocol defined. More precisely, the amount of water and PCM were chosen such that not all the PCM would completely melt but would maintain a constant temperature while solidifying in the cooling down water bath when going below the PCM temperature. In fact, at room temperature in the Netherlands this corresponds to ≈1 L but for example in warmer places the relative amounts might need to be adapted.

LAMP reactions typically work well in the 60–70 °C range, depending on the exact primer sets and enzymes used, and so the device was to be designed such that the LAMP sample vials would be exposed to a temperature of about 65 °C for 25 min. After unsuccessful tests of many household appliances (oven, dishwasher, washing machine, and so on), we devised a working NINA‐LAMP using few grams of PCM, an aluminium coffee capsule, a 3D‐printed holder (Figure 1) and a liter of boiling water. To ensure proper reactions temperature, we selected Rubitherm RT64HC, a paraffin‐based PCM, with a melting temperature between 63 and 65 °C with a main peak of 64 °C. The material is available in large quantities, at low cost (≈13 € kg^−1^). The 5–6 g of PCM and 1 L of water allow for a melting/solidifying process that takes about 25 min. In other words, the amount of PCM was chosen such that it would not completely melt in the hot but slowly cooling down water bath, in such a way it is prevented that the LAMP sample is heated to temperatures higher than the PCM.

The thin aluminum coffee capsules enable efficient heat transfer, and can be produced in large quantities in a short amount of time. The reaction vials are held in place by a polylactic acid (PLA) 3D‐printed vial holder which also can be rapidly produced in large quantities. The holder has been designed to be placed in the top part of the coffee capsule to fit the LAMP‐vials in such a way that the bottom of the vials is more than 3 mm away from the aluminum side of the capsule. This is to avoid the vials getting too close to the aluminum, the temperature may exceed the 64 °C, degrading the enzymes.

### 2.1 T‐Cup Device

To prepare the T‐Cup device, between 5 and 6 g of granular RT64HC were added to a clean coffee capsule. The material was melted by heating the cup by placing it in a hot (close to boiling) water bath. Next, the cup was taken out of the water bath and a 3D printed vial holder was placed in the cup, together with four standard 200 µL PCR tubes. Once the T‐cup was cooled down, the vials are removed, leaving imprints of the vials inside the device. The prepared devices can be stored safely at ambient temperature for months, and probably years, ready for use.

**Figure 2:**
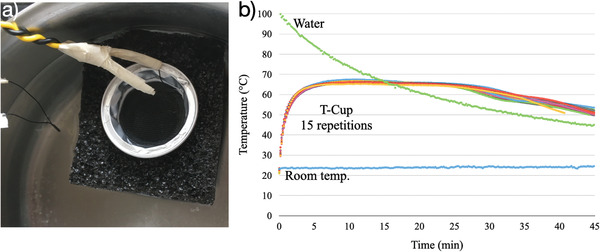
a) Thermistors and T‐Cup used for temperature monitoring. b) Temperature profile of the water bath (green), the room temperature (blue) and the T‐Cup (same capsule for 15 repetitions, repetitions of water temperature, and r.t. removed for clarity of visualization and are presented in Figure S3 of the Supporting Information). Once the heating is stopped the water bath starts decreasing to reach room temperature, while the PCM reaches more than 60 °C in 3 min and manages to keep a temperature between 61 and 67 °C for ≈30 min (Figure S4, Supporting Information). A comparison of the heating curves over time between a commercial LAMP system, a sous vide, and the T‐Cup is depicted in Figure S5 (Supporting Information).

In its use, the T‐Cup device is placed in a pot with boiling water, right after turning off the heating of the pot, enabling rapid increase in temperature of the PCM, stabilizing at ≈64 °C when reaching its melting temperature. To evaluate this, we measured the temperature inside and outside the T‐Cup device using three thermistors coupled to an Arduino microcontroller. This showed that the PCM reached a temperature of more than 60 °C in ≈3 min and maintained a constant temperature between 61 and 67 °C for ≈25 min, sufficient for a typical LAMP reaction (**Figure** **2**). From Figure 2 clearly the buffering capacity of the PCM is illustrated. After the rapid increase in temperature to the PCM temperature, the temperature remains constant whilst the water bath is still at higher temperature until about 15 min after the start of the experiment. After these 15 min, the water bath is at lower temperature than what is required for the LAMP reaction, however, now the PCM is solidifying, releasing energy and keeping the PCM and embedded vials at a constant 64 °C for another ≈10 min before slowly cooling down to room temperature.

#### 2.1.1 Points of Attention

Although the T‐Cup itself floats in water, a stabilizing floating aid is needed to keep the coffee capsule stable. A standard polyurethane or polyethylene foam shipping is more than enough. However, we noticed that when reusing the floating foam, the cold and wet foam slowed down the heating process, poossibly affecting with the LAMP reaction (Figure S1, Supporting Information). The floating foam also helps in not contaminating the T‐Cup with water from the pot, in fact when the PCM was contaminated with water, the temperature was not well stabilized anymore (Figure S2, Supporting Information). We further noticed that inductive heating, compared to fire burner, also keep the temperature too high in the pot, and should be avoided, i.e., once the water is boiling, the water pot should be removed from the inductive heating plate and placed somewhere else. Abovementioned pitfalls should be accounted for in the home‐test procedure when running the LAMP reaction.

### 2.2 LAMP Reaction

In order to validate the thermal stability for running LAMP reactions, we evaluated T‐Cup for LAMP‐based detection of SARS‐CoV‐2 RNA. We used a one‐step RT‐LAMP reaction to perform the reverse transcriptase step, in which viral RNA is converted into a copied DNA, followed by specific LAMP based amplification of target sequence. We used primers for detecting the E‐gene region of the SARS‐CoV‐2 RNA.^[^ ^14^ ^]^ We ran a serial dilution of synthetic RNA from SARS‐CoV‐2 as template.

**Figure 3:**
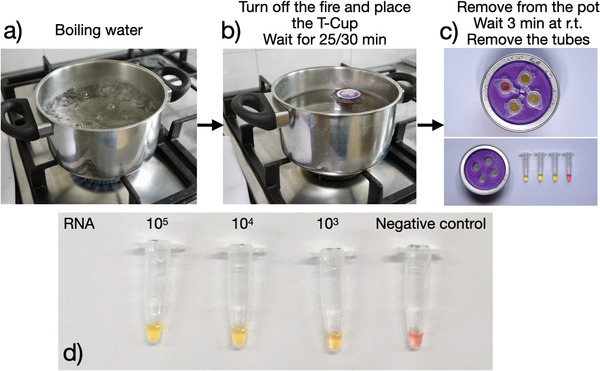
Procedure for the T‐Cup LAMP test. Top: a) turn on the fire and wait for the water to boil. b) Turn the heating off, put the T‐Cup inside and wait for 25/30 min. c) Remove the T‐Cup from the water, let it cool down and remove the vials. d) Example of successful amplification and detection of SARS‐CoV‐2 using the T‐Cup.

The experimental set up is depicted in **Figure** **3**, a pot of water is heated until boiling, after which the heating is turned off and the T‐Cup with the vials in it is added to the pot for 30 min. After removal of the T‐Cup, the cup is allowed to cool down for 3 min, after which the vials are removed and checked for their color. The color of the vial depends on the dye present that changes color depending on the pH which is lowered with a successful LAMP reaction. The colorimetric results show that the reaction was successful, and we managed to detect down to 10^3^ molecules of RNA µL^−1^ in a 25× dilution (using 1 µL 10^3^ µL^−1^ template solution in 25 µL total) using a nonoptimized LAMP reaction. Optimizing the enzymes, the primers and their concentrations for this specific device can improve the sensitivity of the LAMP by at least another order of magnitude, but it is outside of the scope of this research and it should be done anyway for any newly developed LAMP test. We next tested the set up with the analysis of human throat/nasopharyngeal swab samples. For this, RNA extracted from three positively and three negatively tested persons were tested and compared to PCR analyses. All the positives were found positive both in PCR and in the LAMP using the T‐Cup as well as all the negatives were found negative in both analyses (details in the Supporting Information). As the focus of this research was on the development of the device and not the LAMP test itself, this was just a fast proof that the device can run a real‐sample LAMP tests.

At home, once the T‐Cup has been used, the 4 vials can be put in boiling water, >95 °C for 5 min, to deactivate the possible virus and thrown away. The T‐Cup can be reused for consecutive tests once the PCM has reached room temperature again. Considering the reusability of the PCM the real cost of the test boils down to merely the biomaterial and the vials.

Material‐wise, the whole device costs less than 20 Eurocents on the consumer side, probably less than 10 Eurocent on the production side, it can be reused multiple times, and its materials allow for a limited waste production. The 3D‐Printed PLA, in certain conditions, is biodegradable, aluminum (the cup) is one of the best materials to be recycled, with a recycling efficiency close to 100%, and the PCM, composed of paraffin can be burned or repurposed in paraffin rich materials such as candles. Being mostly paraffin, the PCM is also safe to transport, handle, and store.

## 3 Conclusions

We have developed a cheap and simple‐to‐use noninstrumental nucleic acid LAMP device. Considering the design and required materials, it can be produced in millions in short amount of time with already working production in place. The fabrication of such devices is easy and scalable as aluminum cups are produced already in different continents, there are multiple PCM providers, and the 3D printed holder can be done everywhere. With an appropriate assembly line and logistics, it will be easy to produce and ship millions of those devices. It is almost universal, as the only two things one would need to run the test are fire (or electricity) and water. It is also easy to recycle without creating e‐waste or excessive plastic waste. This, for example in contrast with already commercially available kits, like the “Lucira's Covid Test” which costs 89$, is single use (disposable), and produce both plastic waste and e‐waste.^[^ ^15^ ^]^ The T‐Cup device can also be used in low‐ and lower‐income countries, as well as in remote places, or when a large amount of LAMP devices should be deployed as soon as possible. The sample preparation has not been discussed in this paper. Saliva‐based and gargle protocols have been presented that allow collection of virus material by individuals themselves.^[^ ^16^ ^]^ Various commercial and noncommercial extraction free protocols for LAMP have been described in literature.^[^ ^17^ ^]^ This includes both thermal and chemical lysis steps in RNA stabilizing buffers to be used directly in the LAMP assay, as well as cellulose‐based sample prep procedures. The detection method we use for this specific LAMP test is based on a pH dye which may hinder the use of buffer for the lysis steps, this can be overcome by either not using a buffer or by using other detection methods, for example fluorescence, using a simple LED. Obviously, the device as such is not limited to SARS‐CoV‐2 detection but could be employed for any RNA/DNA test with an appropriate set of LAMP primers and enzymes.

## 4 Experimental Section

“How to make it” and “how to use it” guides are provided as documents in the Supporting Information.

The coffee was removed from the aluminium coffee capsules. The capsules were then washed and dried. Rubitherm RT64HC was obtained from Rubitherm GmbH (Germany). For the preparation of the T‐Cup, between 5 and 6 g of RT64HC flakes were placed inside the empty capsule, and the capsule was placed in a pot with boiling water until all the flakes become liquid. Then, the capsule was removed from boiling water and the 3D printed holder was inserted on the top. Four empty PCR tubes were inserted in the holder, and the capsule was left cooling down until room temperature. Once cooled down, the PCR tubes were removed from the T‐Cup. For monitoring the temperatures over time, an Arduino Uno with three thermistors was used as described in ref. [11].

LAMP primers and their concentration for the SARS‐CoV‐2 are described in the Supporting Information.

## Conflict of Interest

The authors declare no conflict of interest.

## Supporting information

Supporting Information

Supporting Information

## Data Availability

The data that support the findings of this study are available in the supplementary material of this article.
