# Supplementary material for: T‐Cup: A Cheap, Rapid, and Simple Home Device for Isothermal Nucleic Acid Amplification
Source: Glob Chall. 2021 Dec 26;6(3):2100078. doi: 10.1002/gch2.202100078 (PMC8902289; doi:10.1002/gch2.202100078)
Supplement: Supplementary file 1 — Supporting Information [file GCH2-6-2100078-s001.pdf]

Global Challenges
Open Access

Supporting Information

for *Global Challenges,* DOI: 10.1002/gch2.202100078

T-Cup: A Cheap, Rapid, and Simple Home Device for Isothermal Nucleic Acid Amplification

*Aldrik H. Velders, Michel Ossendrijver, Bart J. F. Keijser, and Vittorio Saggiomo**

Supplementary material for

T-Cup: a cheap, rapid, and simple home device for isothermal nucleic acid amplification

Aldrik H. Velders,[a] Michel Ossendrijver, Bart J.F. Keijser,[b] Vittorio Saggiomo[a,]*

a) Laboratory of BioNanoTechnology, Wageningen University, PO Box 8038, 6700EK, Wageningen, The Netherlands

b) TNO Microbiology and Systems Biology, PO Box 360, 3700 AJ Zeist, The Netherlands

Email: Vittorio.saggiomo@wur.nl

Figure S1: T-Cup floating support damp with cold water

Figure S2: Water contamination of RT64HC

Figure S3: T-Cup repetitions

Figure S4: Enlargement temperature graphs of T-Cup repetitions

Figura S5: Comparison of temperature curves

Table S1: Evaluation of T-Cup using swab samples

Figure S6: Results from human samples using the T-Cup device.

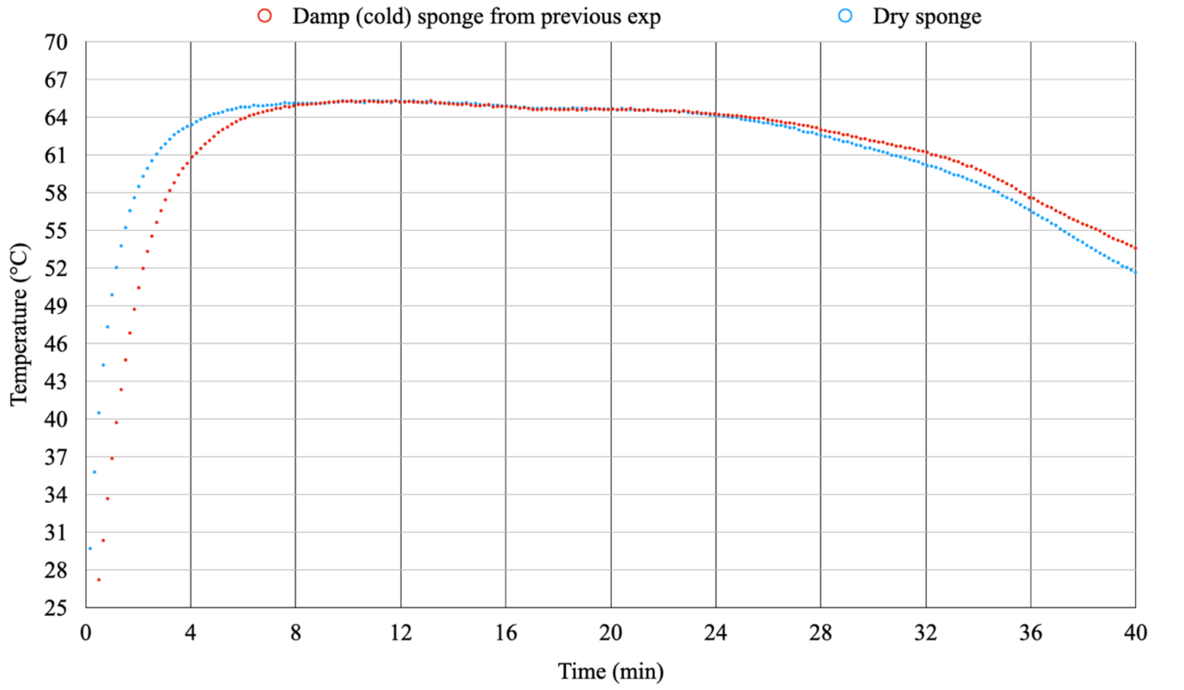

Figure S1: when cold water damp packaging foam is used as floating support for the T-Cup in the hot water, it slows down the heating of the PCM of c.a. 2 minutes.

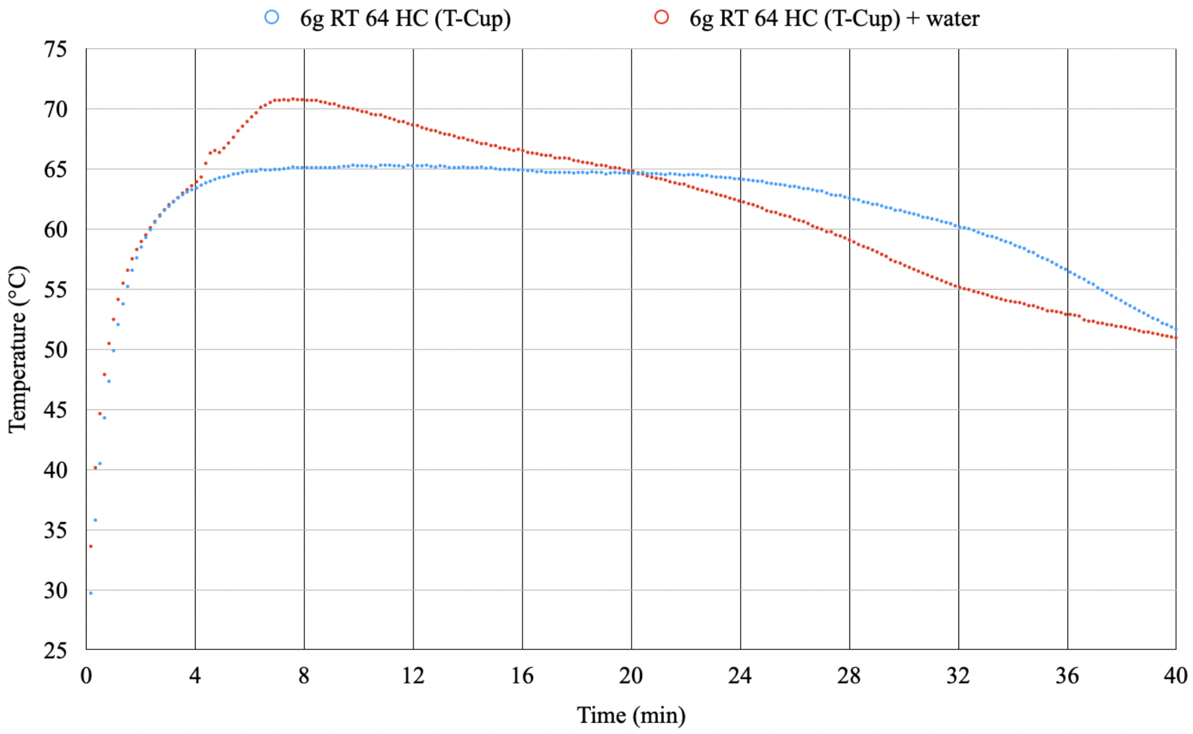

Figure S2: water contamination of the PCM material affect its thermal capabilities.

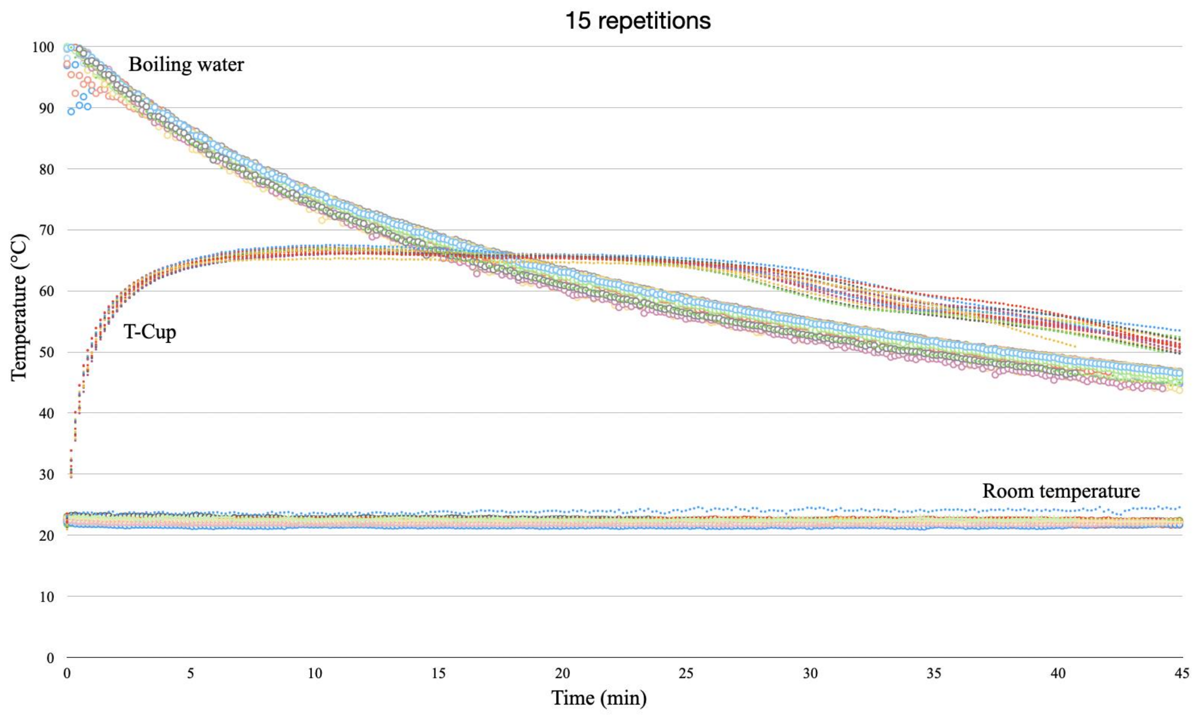

Figure S3: 15 repetitions of a T-Cup experiment. Even when the boiling water is not precisely at 100°C the PCM manages to keep the temperature stable between 61°C and 67°C.

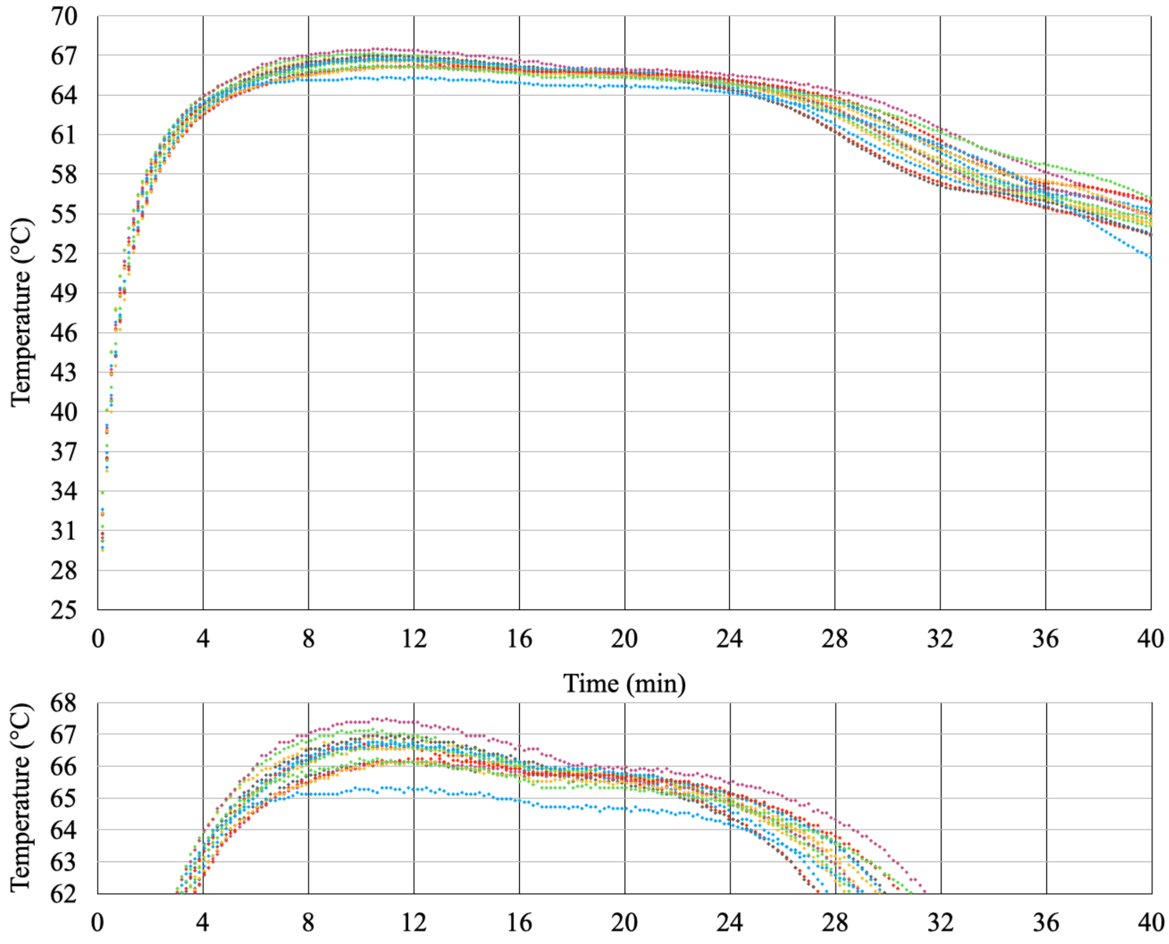

Figure S4: Heating profile of the T-Cup (15 repetitions)

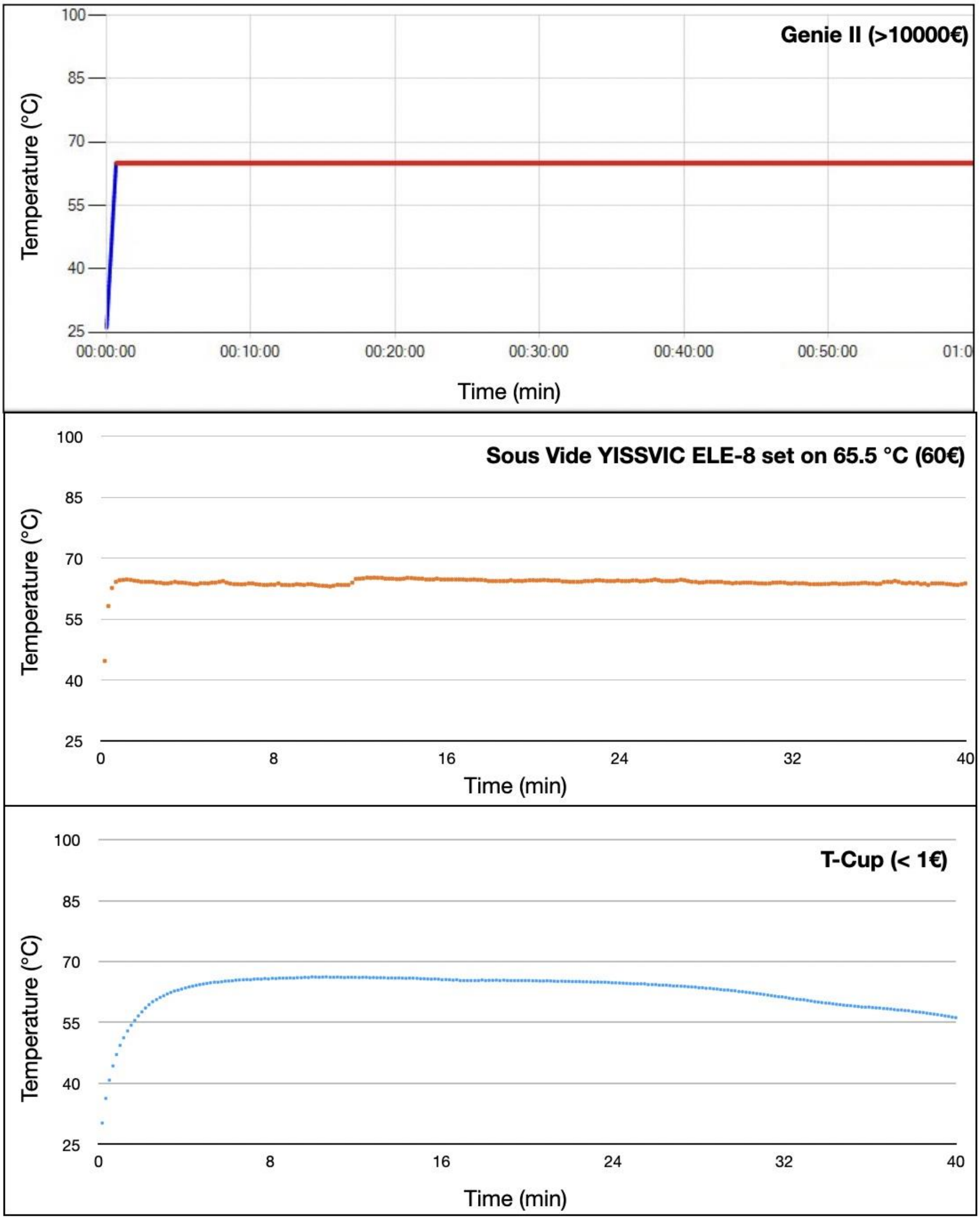

Figure S5. Heating profiles of different systems. From top to bottom: An Optigene Genie II, a Saus Vide, and the T-Cup presented in this research.

Evaluation of T-Cup using swab samples:

To evaluate the T-Cup set-up, RNA extracted from human throat/nasopharyngeal swab samples were obtained at a community testing center (Amsterdam, the Netherlands) from adults with either SARS-CoV2- related symptoms. Participants were informed and consented with participation. For routine SARS CoV-2 qPCR, RNA was isolated using the MagC extraction kit and analysed by PCR using the Allplex™ 2019-nCoV assay (Seegene) in a certified clinical laboratory according to the ISO 15189 standard. Three positively and three negatively tested samples were randomly and anonymously selected for analysis by Rt-LAMP. Positive samples had a Ct value (N-gene) of 26.9, 30.8 and 33.2 respectively. The Colorometric RT-LAMP reactions were set-up according to table S1 using primers targeting the SARS CoV-2 E-gene.[1] We also included a negative template control and a positive template control (synthetic SARS CoV-2 RNA, Twist Bioscience, South San Francisco, USA). T-Cup incubations in hot water were performed as outlined in the protocol. Results were evaluated by visual inspection after 30 minutes of incubation. Positive lamp amplification yielded a change from red to yellow (Figure S6). Full concordance was found with the test results obtained by qPCR.

LAMP protocol for gene E

**RT-LAMP Protocol SARS-CoV2 gene E**		Table 1
Component	***Final Conc.***	***volume per reaction (uL)***
WarmStart® Colorimetric LAMP 2X Master Mix	1X	12.50
FIP Primer (32 uM)	1.6 µM	1.25
BIP Primer (32 uM)	1.6 µM	1.25
F Primer (4 uM)	0.2 µM	1.25
B Primer (4 uM)	0.2 µM	1.25
Loop Primer (8 uM)	0.4 µM	1.25
milli-Q water		5.25
RNA template		1.00
Total Reaction Volume	25 µl	25.00

LAMP primers – E-gene
F3 AGCTGATGAGTACGAACTT
B3 TTCAGATTTTTAACACGAGAGT
FIP ACCACGAAAGCAAGAAAAAGAAGTATTCGTTTCGGAAGAGACAG
BIP TTGCTAGTTACACTAGCCATCCTTAGGTTTTACAAGACTCACGT
LP CTGCGCTTCGATTGTGTGCGT

Table S1. Lamp protocol for the detection of gene E and primers used.

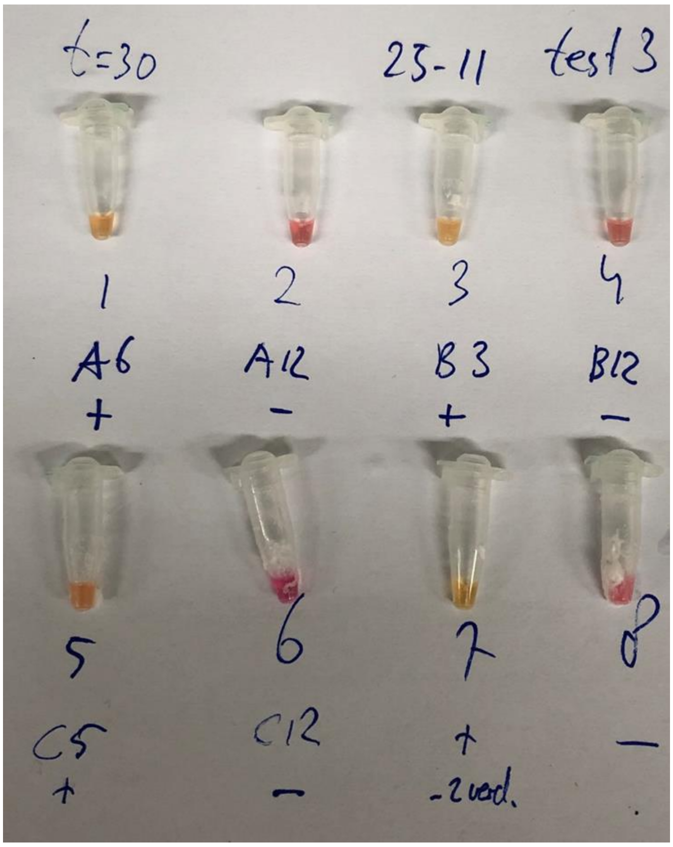

Figure S6. Results from human samples using the T-Cup device.

1) Rapid Detection of SARS-CoV-2 Using Reverse transcription RT-LAMP method
Weihua Yang, Xiaofei Dang, Qingxi Wang, Mingjie Xu, Qianqian Zhao, Yunying Zhou, Huailong Zhao, Li Wang, Yihui Xu, Jun Wang, Shuyi Han, Min Wang, Fenyan Pei, Yunshan Wan
medRxiv 2020.03.02.20030130; doi: https://doi.org/10.1101/2020.03.02.2003013
