# Supplementary material for: T‐Cup: A Cheap, Rapid, and Simple Home Device for Isothermal Nucleic Acid Amplification
Source: Glob Chall. 2021 Dec 26;6(3):2100078. doi: 10.1002/gch2.202100078 (PMC8902289; doi:10.1002/gch2.202100078)
Supplement: Supplementary file 2 — Supporting Information [file GCH2-6-2100078-s002.zip › how to make it.pdf]

How to Make the T-Cup

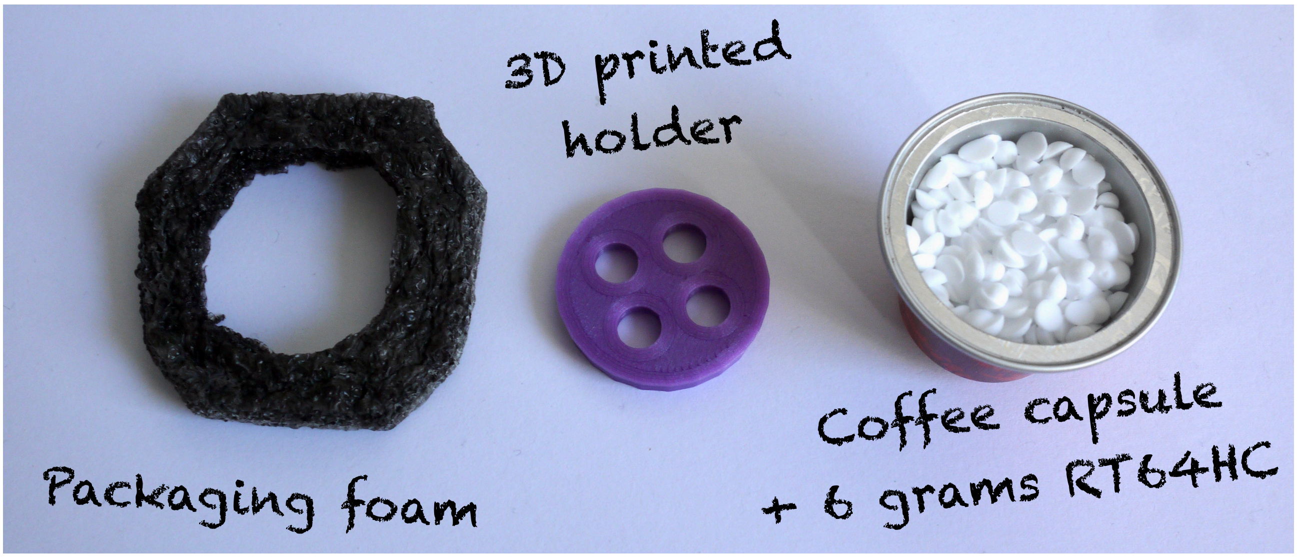

1) Prepare the packaging foam (dry), the 3D printer holder and a clean/ dry coffee capsule with c.a. 6 grams of Rubitherm RT64HC

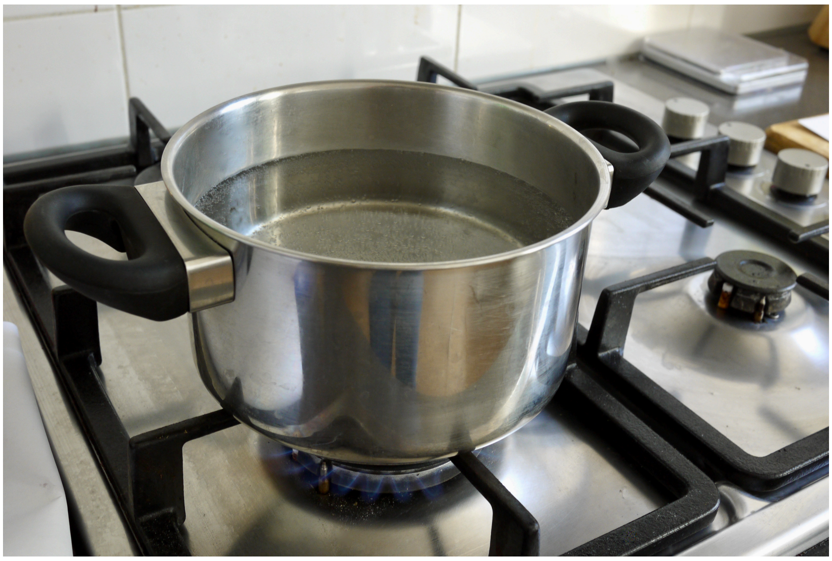

2) Light up the fire under a cooking pot containing water, and gently place the T-Cup in the water using the packaging foam.
Pay attention not to let water inside the T-Cup.

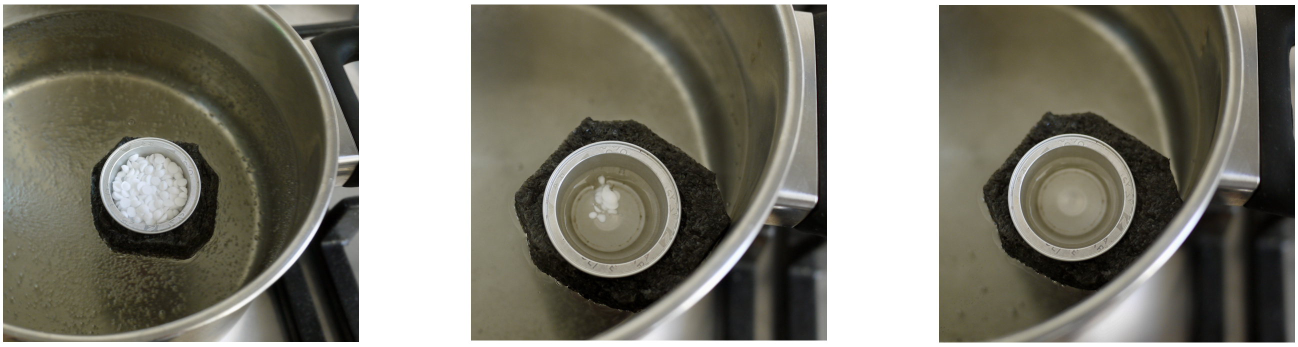

3) While simmering (fire still on on low), wait until all the RT64HC has melted

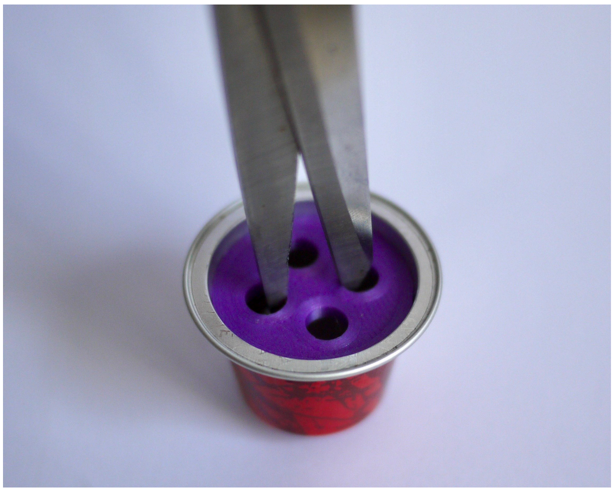

4) When fully melted, remove it from the pot and with the aid of tweezers or scissors place the 3D printed holder in the capsule.
Pay attention! The melted RT64HC is hot!

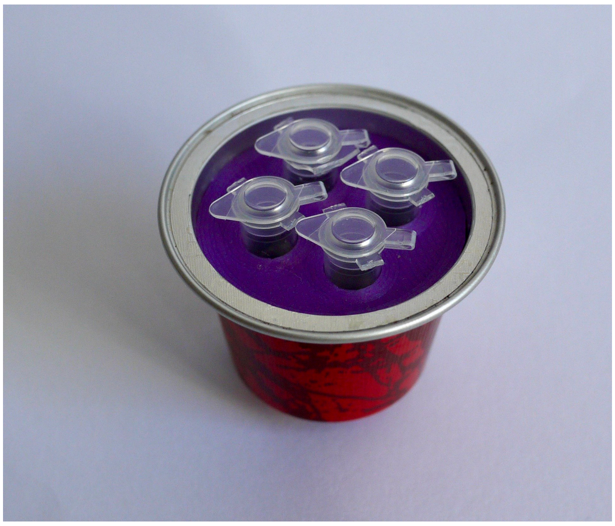

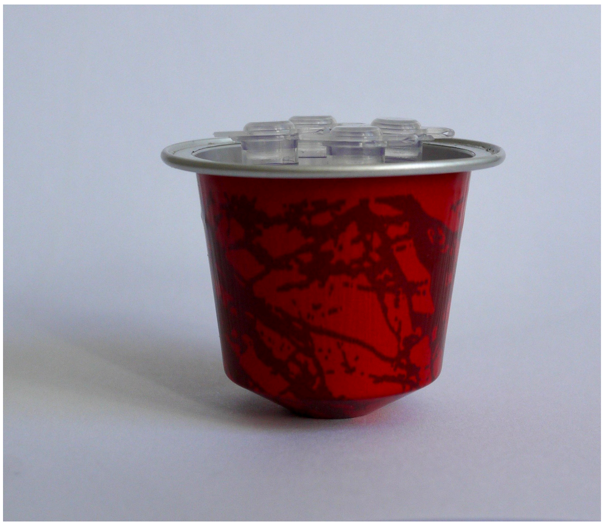

5) Once the 3D printed holder is in place, push it down to fix it.
Then place the PCR tubes inside. They should be 1 or 2 mm higher than the border of the capsule.

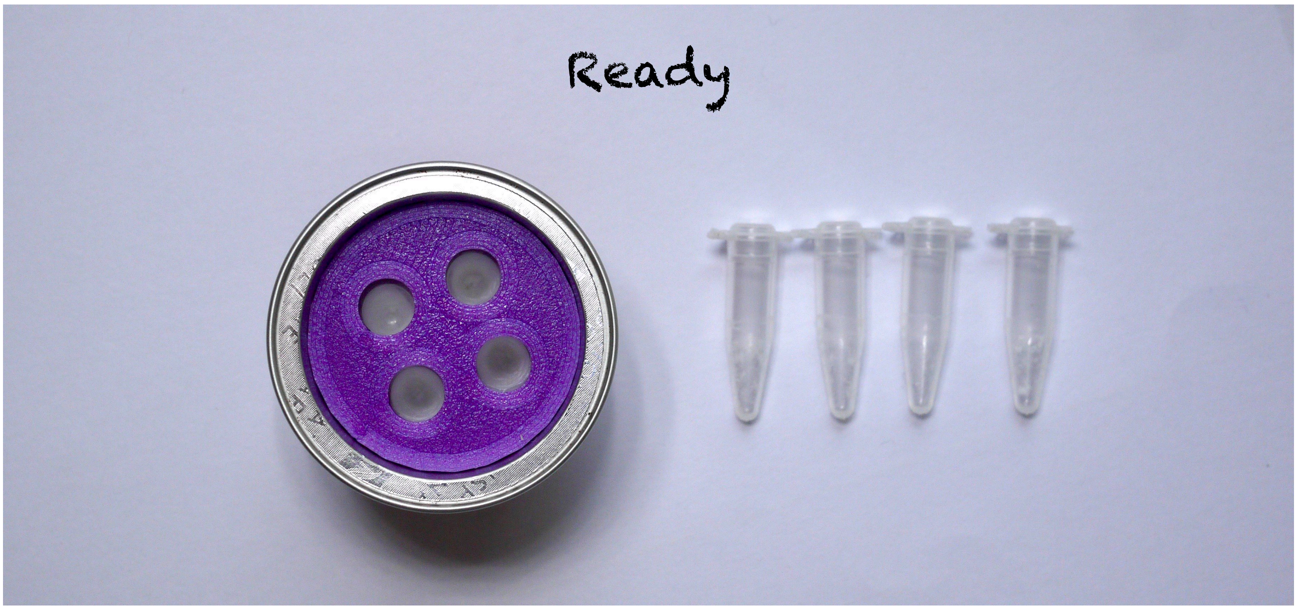

6) Wait until cool (c.a. 1h) then remove the tubes by twisting and pulling them from the device.
Optional but suggested: you can fix the 3D printed holder using glue or by bending the border of the capsule
