# Supplementary material for: T‐Cup: A Cheap, Rapid, and Simple Home Device for Isothermal Nucleic Acid Amplification
Source: Glob Chall. 2021 Dec 26;6(3):2100078. doi: 10.1002/gch2.202100078 (PMC8902289; doi:10.1002/gch2.202100078)
Supplement: Supplementary file 2 — Supporting Information [file GCH2-6-2100078-s002.zip › how to use it.pdf]

How to Use the T-Cup

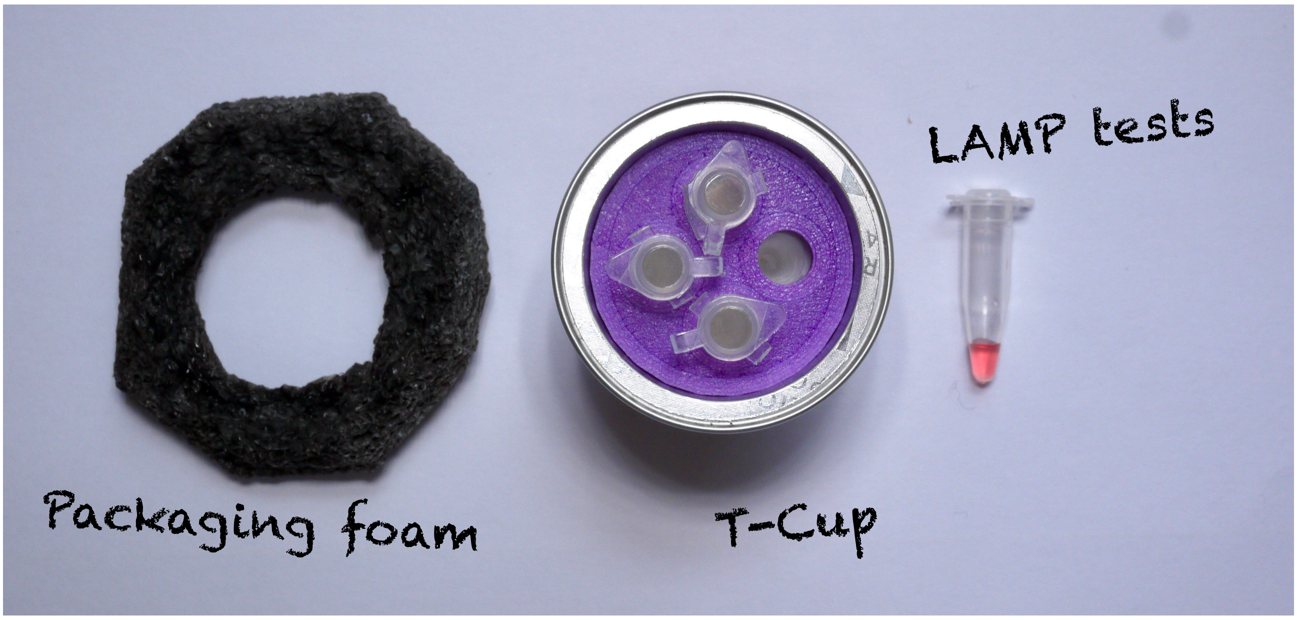

1) Prepare the packaging foam (dry), the 3D printer holder and a clean/ dry coffee capsule with c.a. 6 grams of Rubitherm RT64HC

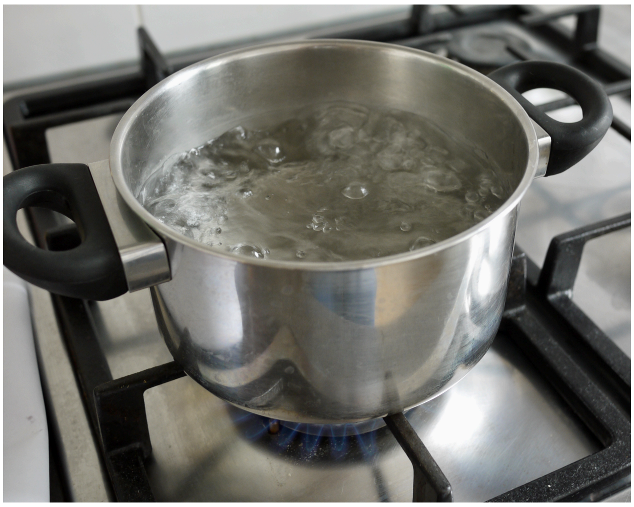

2) Light up the fire and wait until the water (500mL-1L) boil

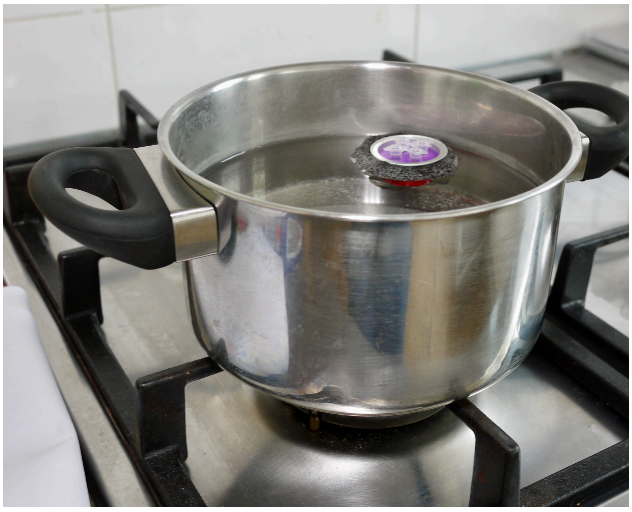

3) Turn off the fire and gently put the T-Cup in the water using the packaging foam.
Pay attention not to let water in the capsule.

In case of electric heating plate, or induction stove, remove the pan from the stove.

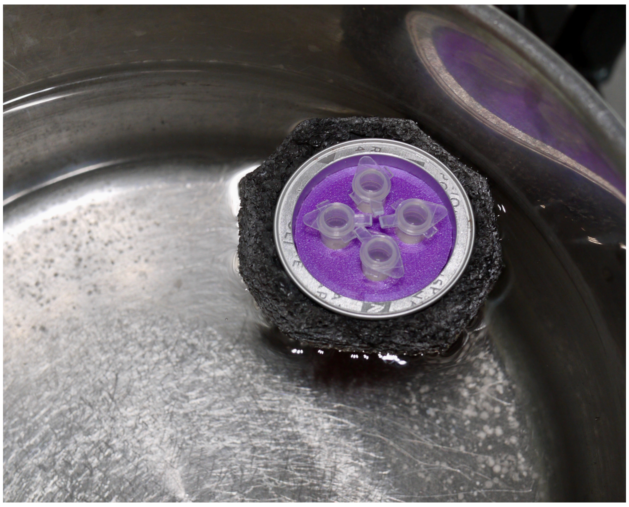

4) Wait for the time necessary (25/30 min depending on the different LAMP assay)

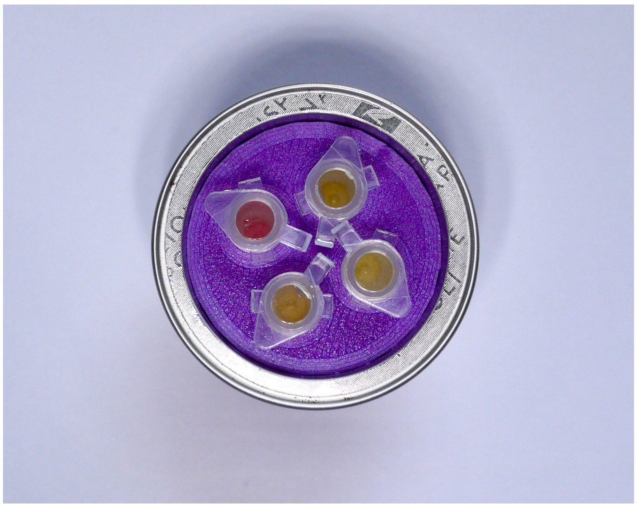

5) Remove the capsule from the cooking pot and wait another 3 min at room temperature

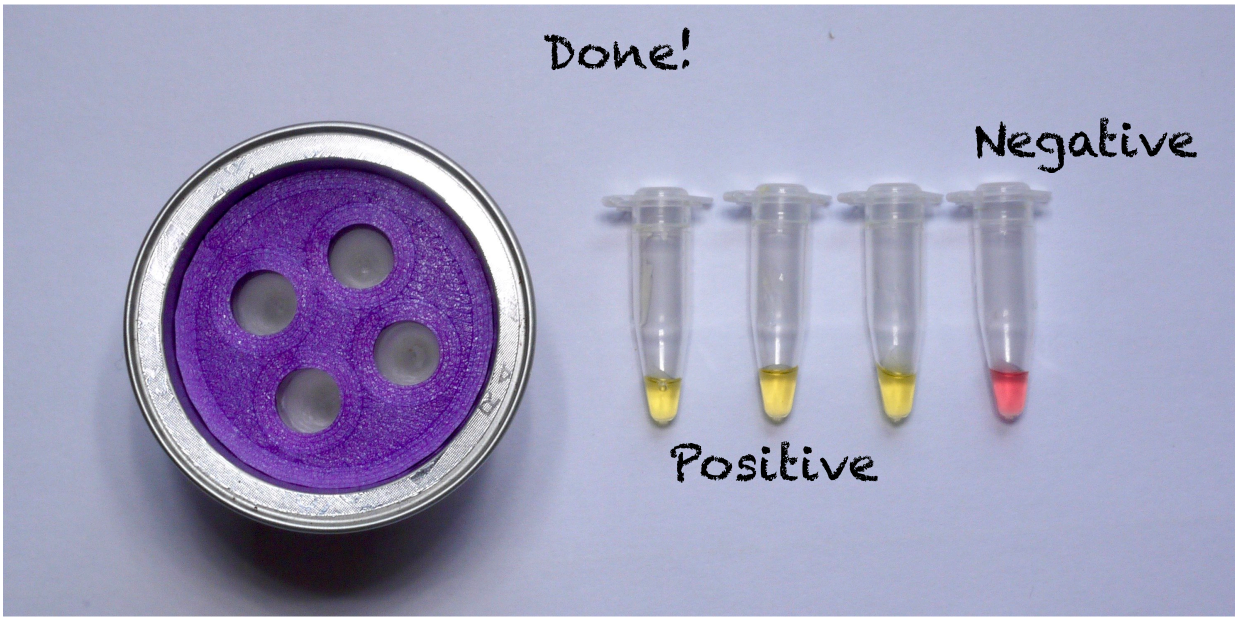

6) Remove the tubes from the T-Cup and read the test.
The T-Cup is reusable, however is should go back on room temperature before a new test is run.
